# Association between Maternal Thyroxine and Risk of Fetal Congenital Heart Defects: A Hospital-Based Cohort Study

**DOI:** 10.1155/2022/3859388

**Published:** 2022-03-11

**Authors:** Jing Dong, Ting Peng, Ming-Qing Li, Feng Xie, Jiang-Nan Wu

**Affiliations:** ^1^Medical Center of Diagnosis and Treatment for Cervical Diseases, Obstetrics and Gynecology Hospital of Fudan University, Shanghai, China; ^2^Department of Obstetrics, Obstetrics and Gynecology Hospital, Fudan University, Shanghai, China; ^3^Shanghai Key Laboratory of Female Reproductive Endocrine-Related Diseases, Shanghai, China; ^4^Department of Clinical Epidemiology, Obstetrics and Gynecology Hospital, Fudan University, Shanghai, China

## Abstract

**Background:**

Evidence for the association between maternal thyroxine concentration and the risk of fetal congenital heart defects (CHDs) is absent. We aimed to study the association of maternal free and total thyroxine (FT4 and TT4) concentrations and the free-to-total thyroxine proportion (FTT4P, %) with the risk of CHD.

**Methods:**

The study was a hospital-based cohort study of 52,047 women who received a universal thyroid function test between 2012 and 2016. CHD was screened by ultrasound between 20 and 24 weeks of gestation or diagnosed until the 42^nd^ day of birth. Adjusted odds ratios (ORs) of fetal CHD were estimated for maternal FT4 and TT4 concentrations or the FTT4P by multivariate logistic regression.

**Results:**

A total of 41,647 women with singleton pregnancies were included for the analysis and 215 CHD cases were detected. The FT4 concentration was significantly associated with a higher risk of CHDs (OR, 1.04, 95% confidence interval (CI): 1.01 to 1.07). Each 1% higher FTT4P was related to a 1.41-fold (95% CI: 0.27 to 3.59) higher risk of CHDs. The association became stronger for women with a thyroid function test performed between 12 and 18 weeks of gestation (OR = 1.05 (95% CI: 1.01 to 1.09) for the FT4 concentration and 3.32 (95% CI: 1.43 to 7.73) for the FTT4P).

**Conclusions:**

A higher FT4 concentration or FTT4P, measured between 12 and 18 weeks of gestation, was associated with an increased risk of CHDs. These findings may provide new insights into the mechanisms of CHDs and evidence for clinical decisions related to thyroid function tests.

## 1. Introduction

Congenital heart defects (CHDs) are the most common birth defects in newborns and an important cause of infant morbidity and mortality [1]. Important progress has been achieved in the understanding of the causes of CHDs over the past decades. Mutations in regulators of heart development during embryogenesis are the major underlying mechanisms of CHDs, while noninherited factors also have effects on the fetal heart, including maternal illness and conditions (e.g., maternal rubella, pregestational diabetes, exposure to thalidomide, or vitamin A cogeners) [2, 3].

Maternal thyroid hormone is essential for fetal growth and development [4, 5]. Maternal thyroid dysfunction is associated with the risk of adverse fetal outcomes, such as fetal growth restriction, preterm birth, and fetal brain underdevelopment [6–9]. Maternal thyroid hormone may regulate key processes in placentation through the secretion of several growth factors and cytokines that are critical for endovascular trophoblast invasion and angiogenesis of maternal and fetal placental vessels [10]. In addition, thyroid hormone might involve in fetal cardiac development [11, 12]. However, evidence for the association between thyroid dysfunction and the risk of CHD is absent.

We conducted a retrospective study to determine the association between thyroid function and the risk of CHD, in which thyroid function was evaluated by free thyroxine (FT4) and total thyroxine (TT4) concentrations since FT4 is a better marker of thyroid function than thyroid-stimulating hormone (TSH) [5, 13, 14], and TT4 measurements are thought to be superior to immunoassay measurements of the FT4 concentration in pregnant women [15, 16]. The free-to-total thyroxine proportion (FTT4P, %), which mainly reflects the status of binding proteins/globulins (e.g., thyroxine-binding globulins, transthyretin, and albumin) and therefore the health status of the mother, was also calculated to estimate its association with the risk of CHD.

## 2. Methods

### 2.1. Study Design and Data Collection

A hospital-based retrospective cohort study was conducted in 2018. Details of the design and methods have been described [17]. Briefly, all pregnant women who received antenatal tests between April 2012 and August 2016 at the hospital were included in the cohort. At their first visit to the hospital, thyroid function was measured, and basic characteristics (e.g., maternal age, location of residence, parity, and model of conception) were recorded. Gestational thyroid disease and complications (such as gestational diabetes and preeclampsia) diagnosed according to the definition of the diseases were extracted from the medical records. Medications used for thyroid diseases (e.g., hyperthyroidism and hypothyroidism) was not collected. Fetal birth defects, including CHD, were confirmed till the 42^nd^ day after the birth. In the present study, women lost to follow-up and those with multiple pregnancies were excluded from the analysis. Women without a TT4 measurement were further excluded when studying the association between TT4/FTT4P and the risk of CHD. The study was approved by the Ethics Committee of the Obstetrics and Gynecology Hospital of Fudan University (No. 2017-35, 2017-35-C1).

### 2.2. CHD Definitions

CHDs were defined as structural abnormalities of the heart or intrathoracic large vasculature. In the present study, fetal CHD cases detected by 20–24 weeks of gestational ultrasound screening and newborns with a CHD diagnosed by sonography or magnetic resonance imaging until the 42^nd^ day of birth [18] were identified as CHD cases. CHD subtypes were coded according to the *International Classification of Disease 10th Revision* (ICD-10) as a ventricular septal defect (Q21.001), atrial septal defect (Q21.102), tetralogy of Fallot (Q21.300), coarctation of the pulmonary artery (Q25.601), truncus arteriosus (Q25.001), common ventricle (Q20.401), dextrocardia (Q24.001), diverticula of the right atrium (Q24.811), patent foramen ovale (Q21.103), and transposition of the great arteries (Q20.301).

### 2.3. Thyroid Function Measurement

Maternal serum samples were collected at the prenatal visits to the hospital (mean visits of 1.5 times, with a range from 1 to 5 times). Thyroid hormone testing, including production of TSH, free and total triiodothyronine (FT3 and TT3), FT4, TT4, thyroperoxidase antibody, thyroglobulin antibody, and TSH receptor antibody, was measured at the first visit, and only three thyroid hormones (TSH, FT3, and FT4) were tested at the subsequent visits. The thyroid function was measured for all subjects included in the analysis at a median gestational age of 15.4 weeks (from 4^+5^ to 39^+6^ weeks).

Thyroid hormone concentrations were measured with an electrochemiluminescence immunoassay (Roche Elecsys, Germany) [17]. Thyroperoxidase, thyroglobulin, and TSH receptor antibodies were regarded as positive if the concentrations were >34 IU/ml, 115 IU/ml, and 1.75 IU/L, respectively. Thyroid antibody positivity was defined if any one of these three markers was determined to be positive. The FTT4P (%) was calculated by a transformation of the concentration unit (pmol/L for FT4 and ng/ml for TT4) and a molecular weight of 776.87 g/mol for thyroxine.

### 2.4. Covariates

Covariates in the study included maternal age at delivery (≤24, 25–34, and ≥35 years), resident location (local or nonlocal), parity (nulliparous or pluriparous), mode of conception (assisted or natural), and gestational diabetes (yes or no), preeclampsia (yes or no), and fetal sex (male or female). Preeclampsia was defined as the onset of hypertension and proteinuria after 20 weeks of gestation [19]. Gestational diabetes was diagnosed based on a 75 g oral glucose tolerance test conducted between the 24^th^ and 28^th^ weeks of gestation [17].

### 2.5. Data Analysis

Continuous variables were expressed as medians (IQR), while categorical variables as numbers (%). The Mann–Whitney U test and the chi-square test or Fisher's exact test (if applicable) were used to compare the differences between the groups of women whose infants had and did not have CHDs. Box diagrams of the distribution of the FT4 concentration and the FTT4P (%) between women with and without a CHD baby according to the gestational age at the thyroid function test (e.g., first, second, or third trimester) were shown.

We constructed logistic regression models to estimate the association of the FT4 and TT4 concentrations and the FTT4P (%) with the risk of CHDs. Adjusted models were further performed to exclude the impact of the covariates mentioned above. Stratified analyses were performed according to thyroid autoantibody status (positive or negative), preeclampsia (yes or no), and time at thyroid function measurement (first or second trimester, or classified by a certain time node, e.g., 12 or 18 weeks of gestation) to test the robustness of the results. Stratification analysis was further conducted according to gestational thyroid disease (e.g., hyperthyroidism or hypothyroidism; yes or no) of pregnant women because the treatment of the disease was unclear in the present study and some antithyroid medications were associated with birth defects [19]. We conducted a sensitivity analysis in which ultrasound-suspected CHD cases were excluded since these cases may be misdiagnosed. The exclusion of women without a TT4 test might induce potential selection bias. We thus constructed missingness analyses by the multiple imputation method [20]. We analyzed the gestational age distribution of the FT4, TT4, and FTT4P. Box diagrams of the distribution of the FT4 and the FTT4P (%) between women with and without a confirmed CHD baby (excluding ultrasound-suspected CHD cases) were further studied according to the gestational age at the thyroid function test (e.g., by trimesters or by a certain time node, e.g., 12 or 18 weeks of gestation). We compared these differences among euthyroid women (i.e., both FT4 and TSH levels between the 2.5^th^ and 97.5^th^ percentiles) to eliminate the potential influence of the outliers on the results.

All analyses were conducted using SPSS (version 21.0, IBM Corp., Armonk, NY, USA). A two-sided value of *P* < 0.05 was considered statistically significant.

## 3. Results

### 3.1. Baseline Characteristics

**Figure 1:**
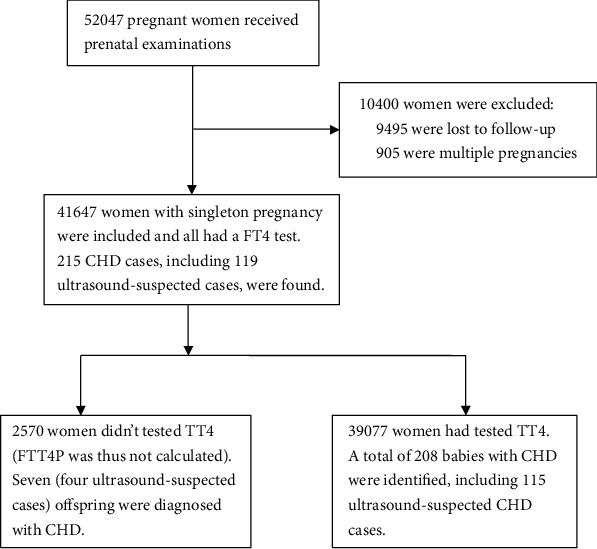
Flow chart of the study.

A total of 52,047 women had visited the hospital, and 41,647 (80.0%) were included for analysis (Figure 1). The basic characteristics were imbalances between women included for analysis and those lost to follow-up. However, no significant differences were detected for the risk of CHD (Table S1). Most of the participants were nulliparous women (84.9%), natural conceptions (98.2%), and local residents (76.9%) (Table 1). During the follow-up period, 215 CHD cases were identified, including 96 newborns diagnosed after birth and 119 fetuses with an ultrasound-suspected CHD. The prevalence of CHD was 5.2 (95% CI: 4.5–5.9) per 1,000 infants, and 2.3 (95% CI: 1.9–2.8) per 1,000 infants for confirmed CHD.

### 3.2. Gestational Thyroxine Change

Figure S1 shows the gestational changes of maternal thyroxine (FT4, TT4, and FTT4P). The FT4 concentration was significantly correlated with the TT4 concentration (Pearson *r* = 0.61, *P* < 0.001). The 12^th^ week of gestation is a critical node; the downward trend of the FT4 before this time accelerates after this point. The tendency for the TT4 concentration before and after this node was different, with a firstly ascended and then began to decrease. The change of the FT4 and TT4 became smaller after 18 weeks of gestation. The gestational week-specific trend of the FTT4P is the same as that of the FT4.

### 3.3. Association of FT4 and FTT4P with the Risk of CHDs

**Figure 2:**
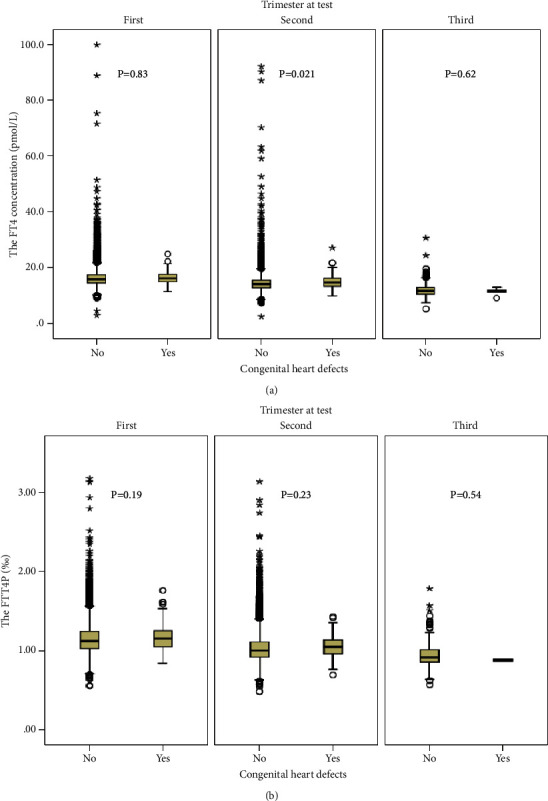
Maternal free thyroxine (pmol/L) and the free-to-total thyroxine proportion (%) between women whose infants had and did not have congenital heart defects by the trimesters at the thyroid function test. Abbreviations: FT4, free thyroxine and FTT4P, free-to-total thyroxine proportion. (a) The free thyroxine and (b) the free-to-total thyroxine proportion. The bottom and top edges of each box represent the first (P25) and third quartiles (P75), respectively. The band within the box represents the median (P50), and the whiskers represent values that are 1.5 times the interquartile range. *P* values for the Mann–Whitney U test.

The FT4 concentration and FTT4P (%) were significantly higher in women with infants with CHD than those whose babies were without CHD (*P* < 0.001 and *P*=0.004, respectively; Table 1). Maternal FT4 concentration in the second trimester for women with a CHD baby was higher than those without a CHD (*P*=0.021) (Figure 2). The FT4 concentration but not the TT4 concentration was significantly associated with a higher risk of CHDs, with an adjusted odds ratio of 1.04 (95% CI: 1.01–1.07). Each 1% higher FTT4P was related to a 1.41-fold (95% CI: 0.27–3.59) higher risk of CHDs (Table 2).

### 3.4. Stratification Analysis

**Figure 3:**
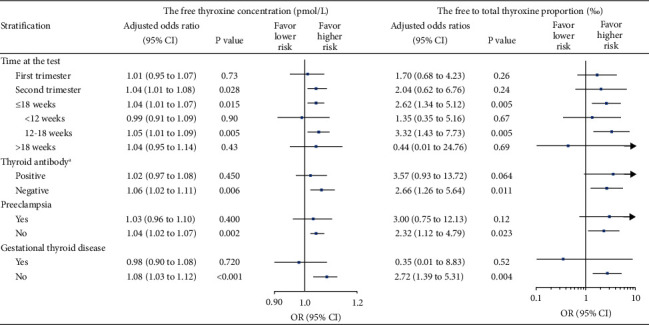
Association of the FT4 and FTT4P with the risk of congenital heart defects in stratification analyses. Abbreviations: FT4, free thyroxine; TT4, total thyroxine; FTT4, free-to-total thyroxine; and 95% CI, 95% confidence interval. ^a^Thyroid antibody positive was defined as any one positive of the three markers, including thyroperoxidase antibody (>34 IU/ml), thyroglobulin antibody (>115 IU/ml), or thyroid-stimulating hormone receptor antibody (>1.75 IU/L).

The associations of the FT4 concentration and the FTT4P with the risk of CHD diagnosed after birth became significant for women with a thyroid function test performed at the 12^th^ week of gestation and decreased from the 18^th^ week of gestation (Figure S2). These disparities were found in the stratification by the time at the thyroid function test. The strongest association was identified when we restrict to those who had a thyroid test between 12 and 18 weeks of gestation (OR were 1.05 and 3.32, respectively; Figure 3). Statistical significant associations were found in women negative for thyroid autoantibodies and those without preeclampsia or thyroid disease complications during pregnancy (Figure 3).

### 3.5. Sensitivity Analysis

In the sensitivity analysis that we excluded the ultrasound-suspected CHD cases, more obvious differences and stronger associations were found, especially for the FTT4P. The FTT4P was higher in women who gave birth to a CHD infant than those without a CHD birth in the first and second trimesters (*P*=0.016 and 0.010, respectively; Figure S3). Higher FTT4P was related to an increased risk of CHD, with an adjusted odds ratio of 4.34 (95% CI: 1.92–9.79; Table S2). The association strengths between FTT4P and risk of CHDs were consistent across the multiple imputation groups, with adjusted ORs ranging from 4.08 to 4.96 (all *P* ≤ 0.001; Table S3). The differences in the FT4 concentration and FTT4P between women with and without a CHD offspring got greater when the thyroid function test was performed between 12 and 18 weeks of gestation (Figure S4). Similar results were detected when we restricted to euthyroid women (Figures S5 and S6).

## 4. Discussion

In the present study, higher maternal FT4 concentration was associated with an increased risk of CHDs. In addition, The FTT4P was a better marker than the FT4 in relating to the risk of CHDs, especially for confirmed CHD risk that diagnosed after birth. The associations were stronger for women with a thyroid function test performed between 12 and 18 weeks of gestation.

To our knowledge, this is the first study that investigates the relationship between thyroid function and the risk of CHDs. Maternal FT4 is transported to the fetus and has a persistent effect on the fetal compartment [13]. The fetal thyroid gland is not functionally mature until 18 to 20 weeks of gestation. Thus, maternal FT4 is critical for fetal growth and development [5]. In addition, maternal FT4 is involved in placentation [4, 13]. A high FT4 concentration might lead to placental function impairment that may further affect fetal nutrient supply and metabolic waste product elimination [13, 21]. Therefore, the FT4 concentration was associated with fetal brain underdevelopment, fetal growth restriction, and preterm birth [6–9]. The present study extends the limited knowledge of the impact of maternal FT4 on the risk of CHDs.

TT4 levels are more stable during pregnancy than FT4 concentrations and better reflect changes in the hypothalamic-pituitary-thyroid axis [15, 16]. However, more than 99% of TT4 is protein-bound, and TT4 is either not associated or not better associated with adverse fetal outcomes than FT4 [5, 14]. In the present study, the TT4 concentration trend during pregnancy differed from the previous opinion that TT4 would continue to rise and reach a peak by approximately week 16 of gestation [5]. Although no significant associations were found between TT4 concentrations and the risk of CHDs, the FTT4P derived from the FT4 and TT4 concentrations was better related to the risk of CHDs than the FT4 concentration. The FT4 immunoassay concentration may be influenced by increased TBG and decreased albumin during pregnancy [22]. The FTT4P might accurately assess maternal thyroid status and reflects the status of binding proteins and globulins during pregnancy, as well as the health status of the mother. These advantages might explain the disparity in their association with CHDs.

Maternal thyroid thyroxine has a more persistent effect on the fetal side and may regulate angiogenic factors and cytokine secretion that are involved in fetal angiogenesis and vascular development [10, 13, 23]. These might largely explain the association of the FT4 concentration and FTT4P with the risk of CHDs. Furthermore, a high maternal FT4 concentration may impair placental function [13], leading to a placental-mediated effect on the risk of CHDs, which might partially explain the association. The association became strong when we restricted to women with a thyroid function test performed between 12 and 18 weeks of gestation when both the FT4 and TT4 concentrations exhibited novel changes. The specific findings might indicate that fetal heart development during this period might be more sensitive to the effect of maternal thyroxine. Early steps in heart development are completed within 5 weeks of gestation, while fetal heart maturation begins from 50 days of human embryo birth [24]. Thus, maternal thyroxine might, neither in a direct nor in an indirect method or both, affect fetal heart maturation, including the septation of ventricular, atrial, and outflow tracts.

We identified gestational week-specific associations in the present study. These novel results indicated that maternal thyroxine or its underlying regulatory mechanisms (e.g., the hypothalamic-pituitary-thyroid axis) might partially impede the maturation of the fetal heart. Making clear the reasons of the specific might help better understand the mechanisms of fetal heart maturation.

Universal thyroid function evaluations are not recommended for pregnant women [16]. The results of the present study do not suggest changing this since the cost might be high in the context of a rare prevalence of fetal CHDs. However, a 12–18-week gestational thyroxine test may be an option for those at higher risk of fetal CHDs and a beneficial supplement for further CHD ultrasound screening. Moreover, antithyroid drug exposure is related to a higher risk of fetal birth defects, while its association with CHD is still unknown. It is hard to distinguish whether the consequence of drug exposure is due to drugs themselves or the high maternal thyroxine. However, there might be an overestimation of the antithyroid drugs on the risk of birth defects in previous studies, in which the harm of high thyroxine might be ignored.

Our study has several limitations. First, the prevalence of infantile CHDs in the present study was lower, especially for confirmed CHD cases diagnosed after birth. This underestimation may be related to the women lost to follow-up (the lost to follow-up rate was 18.2%) who may have a higher prevalence of congenital malformations [25]. The low rate of infantile CHDs may partially be related to the decline of CHD due to the free folic acid supplement program for pregnant women [26]. However, we do not think the strong association would be significantly affected by the potential underestimation of CHDs. Second, the first-visit measurements of FT4 and TT4 were selected for the analysis since only 13,965 women had received the measurement twice, and the follow-up measurements only involved three markers (TSH, FT4, and FT3). We were thus not able to assess the changes in the FT4 concentration and FTT4P and evaluate their impact on the risk of CHDs. Furthermore, the reliability of the FT4 assay, which was less reliable than the TT4 during pregnancy, might influence the stability of the results.

Some risk factors for CHDs [27], such as maternal chronic conditions and habits, history of adverse outcomes in previous pregnancies, and the treatment for gestational thyroid disease, were not collected in the present study. However, we do not think the lack of these factors in the present study would significantly shake the results since the population-attributable fraction of these factors was low (1.2%), the large sample size in the present study had enough power to ensure robust associations, and pregnant women rarely smoke (<1.0%) in Shanghai, China [28]. Finally, although the findings were powered by a large sample of women and the missingness analyses showed consistent results, we acknowledge a potential selection bias induced by a certain percentage of women who were lost to follow-up. The present study was a single-center study in Shanghai where the iodine status of pregnant women was iodine deficient [29]. Most of the CHD cases in this study were septation defects (90 of 96, 93.8%). In addition, antithyroid drug exposure is associated with a higher risk of fetal birth defects. However, we cannot rule out this impact on our results since there was a lack of data on thyroid treatment. Therefore, multiple-center studies are warranted to identify the generalizability of our results.

## 5. Conclusions

In this cohort study of a large number of Chinese pregnant women, a higher FT4 concentration or FTT4P, measured between 12 and 18 weeks of gestation, was associated with the risk of CHDs. These findings may help better understand thyroid function changes during pregnancy, provide new insights into the mechanisms of CHD, and provide evidence for clinical decisions related to thyroid function tests and fetal CHD screening.

## Figures and Tables

**Table 1:** Basic characteristics of the population.

Characteristics	Congenital heart defects		*P* values^a^
	No (*N* = 41,432)	Yes (*N* = 215)	
FT4 (pmol/L, median, IQR)	14.7 (13.2–16.4)	15.6 (13.8–17.1)	<0.001
TT4 (ng/ml, median, IQR)^b^	11.1 (9.8–12.4)	11.1 (9.9–12.4)	0.92
FTT4P (%, median, IQR)^b,c^	1.05 (0.95–1.17)	1.09 (0.99–1.20)	0.004
Antibody positivity^b^ (*n*, %)	4,885 (13.9)	30 (15.7)	0.20
Maternal age (years; *n*, %)			
<25	2,100 (5.1)	10 (4.7)	0.31
25–34	35,231 (85.0)	177 (82.3)	
≥35	4,101 (9.9)	28 (13.0)	
Local residents (*n*, %)	31,866 (76.9)	164 (76.3)	0.83
Nulliparous women (*n*, %)	35,153 (84.8)	188 (87.4)	0.29
Assisted conceptions (*n*, %)	747 (1.8)	6 (2.8)	0.28
Gestational diabetes (*n*, %)	3,478 (8.4)	23 (10.7)	0.23
Preeclampsia (*n*, %)	2,337 (5.6)	31 (14.4)	<0.001
Male fetuses (*n*, %)	21,345 (51.5)	118 (45.8)	0.33

Abbreviations: IQR, interquartile range; FT4, free thyroxine; TT4, total thyroxine; and FTT4P, free-to-total thyroxine proportion. ^a^*P* values for Mann–Whitney U tests or chi-square/Fisher's exact tests. ^b^A total of 39,077 women (38,984 women with a non-CHD baby and 93 women with a CHD offspring) had measured TT4 and an FTT4 *P* value. 35,339 women had tested thyroid antibody, including 35,256 women without a CHD baby. ^c^Molecular weight of 776.87 g/mol for thyroxine was used for the transformation.

**Table 2:** Odds ratios for the risk of congenital heart defects.

Thyroxine	Unadjusted models		Adjusted models^a^	
	Odds ratio (95% CI)	*P* value	Odds ratio (95% CI)	*P* value
FT4 (pmol/L)	1.04 (1.02–1.07)	0.001	1.04 (1.01–1.07)	0.003
TT4 (ng/ml)	1.01 (0.94–1.08)	0.85	1.01 (0.95–1.08)	0.78
FTT4P (%)	2.59 (1.35–4.97)	0.004	2.41 (1.27–4.59)	0.007

Abbreviations: FT4, free thyroxine; TT4, total thyroxine; FTT4P, free-to-total thyroxine proportion; and 95% CI, 95% confidence interval. ^a^Adjusted factors included maternal age (<25, 25–34, or ≥35), residence (local or nonlocal), parity (nulliparous or pluriparous), assisted conception (yes or no), gestational diabetes (yes or no), preeclampsia (yes or no), and fetal sex (male or female).

## Data Availability

The data sets used in the present study are available from the corresponding author (wjnhmm@126.com) on reasonable request.

## Abbreviations

CHD: Congenital heart defect
CI: Confidence interval
FT4: Free thyroxine
FTT4P: Free-to-total thyroxine proportion
OR: Odds ratio
TSH: Thyroid-stimulating hormone
TT4: Total thyroxine.

## Supplementary Materials

Supplementary Materials
*Table S1.* The outcomes and baseline characteristics between women with and without measurements of the TT4/FTT4P. *Table S2.* Odds ratios for the risk of congenital heart defects diagnosed after birth. *Table S3.* Association of TT4 and FTT4P with risk of congenital heart defects diagnosed after birth after multiple imputations for missing data. *Figure S1.* The FT4 and TT4 concentrations and the FTT4P trends (mean and 95% confidence interval) by a week of gestation at test. The solid point on the curve represents the mean of thyroid hormone for women tested at the gestational week. Abbreviations: FT4, free thyroxine; TT4, total thyroxine; and FTT4P, free-to-total thyroxine proportion. *Figure S2.* Odds ratios for congenital heart defects diagnosed after birth by gestational age at the thyroid function test. Abbreviations: FT4, free thyroxine and FTT4P, free-to-total thyroxine proportion. FT4 (panel A) and FTT4P (panel B) are shown. *Figure S3.* Maternal free thyroxine (pmol/L) and the free-to-total thyroxine proportion (%) between women whose infants had and did not have congenital heart defects diagnosed after birth by the trimesters at the thyroid function test. *P* values for the Mann–Whitney U test. *Figure S4.* The maternal FT4 concentration and FTT4P during pregnancy between women with newborns with and without complicated congenital heart defects by gestational age at the thyroid function test (<12, 12–18, or >18 weeks). Abbreviations: FT4, free thyroxine and FTT4P, free-to-total thyroxine proportion. FT4 (panel A) and FTT4P (panel B) are shown. *P* values for the Mann–Whitney U test. *Figure S5.* The maternal FT4 concentration and FTT4P during pregnancy between euthyroid women with and without newborns complicated congenital heart defects at trimesters (first, second, or third). Abbreviations: FT4, free thyroxine and FTT4P, free-to-total thyroxine proportion. FT4 (panel A) and FTT4P (panel B) are shown. *P* values for the Mann–Whitney U test. *Figure S6.* The maternal FT4 concentration and FTT4P during pregnancy between euthyroid women with and without newborns complicated congenital heart defects by gestational age at the thyroid function test (<12, 12–18, or >18 weeks). Abbreviations: FT4, free thyroxine and FTT4P, free-to-total thyroxine proportion. FT4 (panel A) and FTT4P (panel B) are shown. *P* values for the Mann–Whitney U test.
