# Supplementary material for: Association between Maternal Thyroxine and Risk of Fetal Congenital Heart Defects: A Hospital-Based Cohort Study
Source: Int J Endocrinol. 2022 Mar 11;2022:3859388. doi: 10.1155/2022/3859388 (PMC8933103; doi:10.1155/2022/3859388)
Supplement: Supplementary Materials — Table S1. The outcomes and baseline characteristics between women with and without measurements of the TT4/FTT4P. Table S2. Odds ratios for the risk of congenital heart defects diagnosed after birth. Table S3. Association of TT4 and FTT4P with risk of congenital heart defects diagnosed after birth after multiple imputations for missing data. Figure S1. The FT4 and TT4 concentrations and the FTT4P trends (mean and 95% confidence interval) by a week of gestation at test. The solid point on the curve represents the mean of thyroid hormone for women tested at the gestational week. Abbreviations: FT4, free thyroxine; TT4, total thyroxine; and FTT4P, free-to-total thyroxine proportion. Figure S2. Odds ratios for congenital heart defects diagnosed after birth by gestational age at the thyroid function test. Abbreviations: FT4, free thyroxine and FTT4P, free-to-total thyroxine proportion. FT4 (panel A) and FTT4P (panel B) are shown. Figure S3. Maternal free thyroxine (pmol/L) and the free-to-total thyroxine proportion (%) between women whose infants had and did not have congenital heart defects diagnosed after birth by the trimesters at the thyroid function test. P values for the Mann–Whitney U test. Figure S4. The maternal FT4 concentration and FTT4P during pregnancy between women with newborns with and without complicated congenital heart defects by gestational age at the thyroid function test (<12, 12–18, or >18 weeks). Abbreviations: FT4, free thyroxine and FTT4P, free-to-total thyroxine proportion. FT4 (panel A) and FTT4P (panel B) are shown. P values for the Mann–Whitney U test. Figure S5. The maternal FT4 concentration and FTT4P during pregnancy between euthyroid women with and without newborns complicated congenital heart defects at trimesters (first, second, or third). Abbreviations: FT4, free thyroxine and FTT4P, free-to-total thyroxine proportion. FT4 (panel A) and FTT4P (panel B) are shown. P values for the Mann–Whitney U test. Figure S6. The maternal FT4 concen [file 3859388.f1.docx]

Table S1. The outcomes and baseline characteristics between women with and without measurements of the TT4/FTT4P.

| Outcomes and characteristics | | TT4/FTT4P available | | P value |
| --- | --- | --- | --- | --- |
|  |  | Yes (N=39077) | No (N=2570) |  |
| Congenital heart defects | | 93 (0.2) | 3 (0.1) | 0.214 |
| Maternal age (years) | |  |  | 0.651 |
|  | < 25 | 1989 (5.1) | 121 (4.7) |  |
|  | 25-34 | 33220 (85.0) | 2188 (85.1) |  |
|  | ≥ 35 | 3868 (9.9) | 261 (10.2) |  |
| Local residents | | 29914 (76.6) | 2116 (82.3) | <0.001 |
| Nulliparous | | 33112 (84.7) | 2229 (86.7) | 0.006 |
| Assisted conception | | 699 (1.8) | 54 (2.1) | 0.250 |
| Gestational diabetes | | 3328 (8.5) | 173 (6.7) | 0.002 |
| Preeclampsia | | 2242 (5.7) | 126 (4.9) | 0.077 |
| Male fetuses | | 20128 (51.5) | 1335 (51.9) | 0.668 |

Abbreviations: TT4, total thyroxine; FTT4P, free to total thyroxine proportion.

Table S2. Odds ratios for the risk of congenital heart defects diagnosed after birth

| Thyroxine | Unadjusted　models | | Adjusted models ^a^ | |
| --- | --- | --- | --- | --- |
|  | Odds ratio (95%CI) | P value | Odds ratio (95%CI) | P value |
| FT4 (pmol/L) | 1.05 (1.02-1.08) | <0.001 | 1.05 (1.02-1.08) | 0.002 |
| TT4 (ng/ml) | 0.99 (0.90-1.09) | 0.85 | 1.00 (0.91-1.10) | 0.98 |
| FTT4P (‰) | 4.90 (2.15-11.14) | <0.001 | 4.34 (1.92-9.79) | <0.001 |

Abbreviations: FT4, free thyroxine; TT4, total thyroxine; FTT4P, free to total thyroxine proportion; 95% CI, 95% confidence interval.

^a^ Adjusted factors included maternal age (<25, 25-34, or ≥ 35), residence (local or nonlocal), parity (nulliparous or pluriparous), assisted conception (yes or no), gestational diabetes (yes or no), preeclampsia (yes or no) and fetal sex (male or female).

Table S3. Association of TT4 and FTT4P with risk of congenital heart defects diagnosed after birth after multiple imputations for missing data

| Multiple imputation number | TT4 (ng/ml) | | FTT4P (‰) | |
| --- | --- | --- | --- | --- |
|  | Adjusted odds ratio (95% CI) | P value | Adjusted odds ratio (95% CI) | P value |
| 1 | 1.001 (0.909-1.102) | 0.981 | 4.467 (2.020-9.876) | <0.001 |
| 2 | 0.998 (0.906-1.099) | 0.972 | 4.077 (1.808-9.191) | 0.001 |
| 3 | 0.999 (0.907-1.100) | 0.986 | 4.212 (1.880-9.435) | <0.001 |
| 4 | 1.005 (0.913-1.106) | 0.925 | 4.388 (1.980-9.724) | <0.001 |
| 5 | 1.009 (0.917-1.110) | 0.859 | 4.957 (2.297-10.694) | <0.001 |
| Pooled | 1.002 (0.910-1.104) | 0.962 | 4.410 (1.958-9.932) | <0.001 |
| Original data | 1.001 (0.908-1.104) | 0.980 | 4.337 (1.921-9.789) | <0.001 |

Abbreviations: TT4, total thyroxine; FTT4P, free to total thyroxine proportion; 95% CI, 95% confidence interval.

Adjusted factors included maternal age (<25, 25-34, or ≥ 35), residence (local or nonlocal), parity (nulliparous or pluriparous), assisted conception (yes or no), gestational diabetes (yes or no), preeclampsia (yes or no) and fetal sex (male or female).

Figure S1. The FT4 and TT4 concentrations and the FTT4P trends (mean and 95%confidence interval) by week of gestation at test

The solid point on the curve represents the mean of thyroid hormone for women tested at the gestational week.

Abbreviations: FT4, free thyroxine; TT4, total thyroxine; FTT4P, free to total thyroxine proportion.

FTT4P

Figure S2. Odds ratios for congenital heart defects diagnosed after birth by gestational age at the thyroid function test.

Abbreviations: FT4, free thyroxine; FTT4P, free to total thyroxine proportion.

FT4 (panel A) and FTT4P (panel B) are shown.


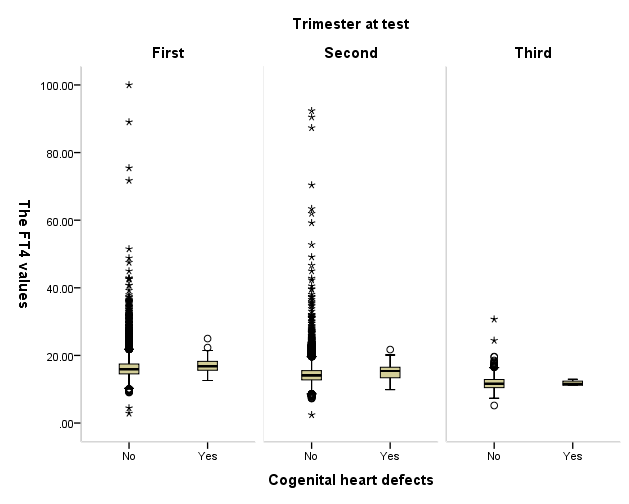

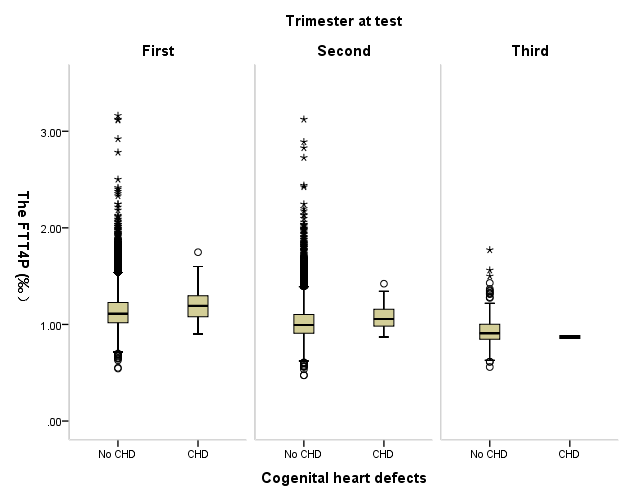


**B**

**A**

P=0.537

P=0.010

P=0.016

P=0.936

P=0.012

P=0.087

Figure S3. Maternal free thyroxine (pmol/L) and the free to total thyroxine proportion (‰) between women whose infants had and did not have congenital heart defects diagnosed after birth by the trimesters at the thyroid function test.

P values for the Mann-Whitney U test.


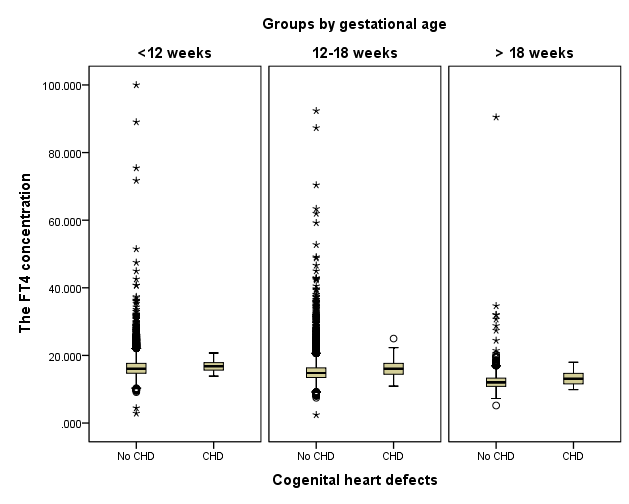


A

P=0.089

P<0.001

P=0.134


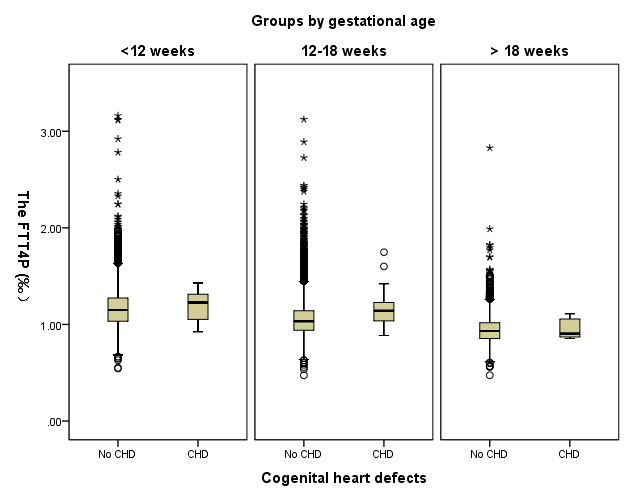


B

P=0.900

P < 0.001

P=0.384

Figure S4 The maternal FT4 concentration and FTT4P during pregnancy between women with and without newborns complicated congenital heart defects by gestational age at the thyroid function test (<12, 12-18, or >18 weeks).

Abbreviations: FT4, free thyroxine; FTT4P, free to total thyroxine proportion.

FT4 (panel A) and FTT4P (panel B) are shown.

P values for the Mann-Whitney U test.


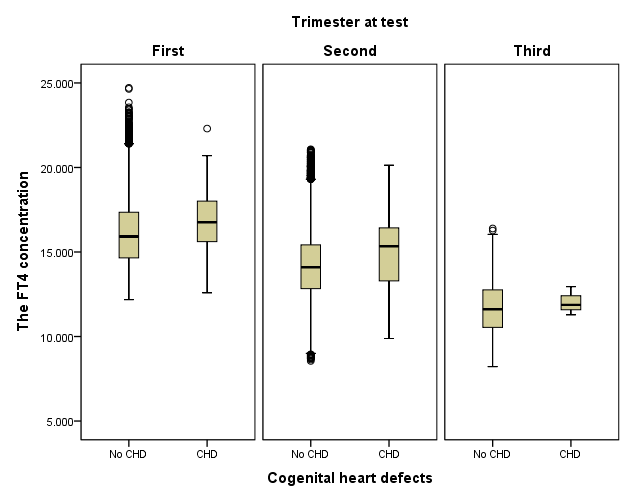

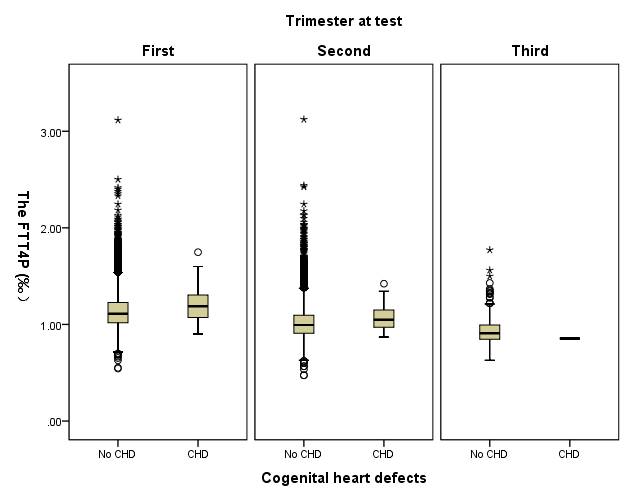


P=0.441

P=0.577

P=0.006

P=0.005

P=0.005

P=0.025

B

A

Figure S5. The maternal FT4 concentration and FTT4P during pregnancy between euthyroid women with and without newborns complicated congenital heart defects at trimesters (first, second, or third).

Abbreviations: FT4, free thyroxine; FTT4P, free to total thyroxine proportion.

FT4 (panel A) and FTT4P (panel B) are shown.

P values for the Mann-Whitney U test.


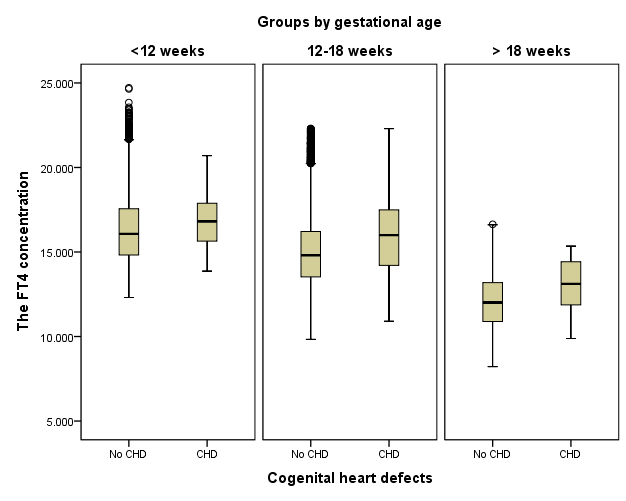

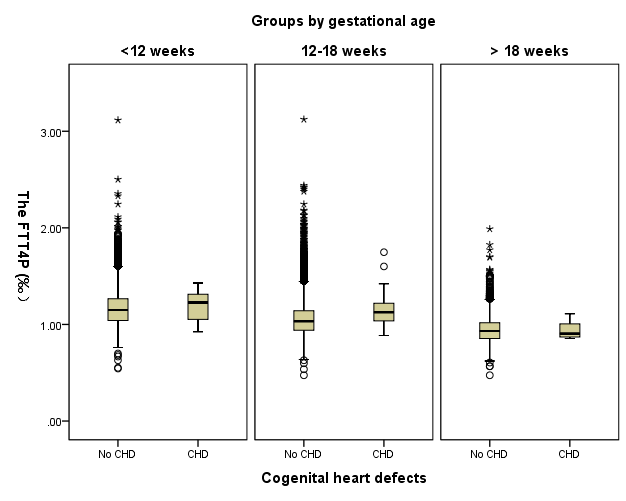


B

P=0.968

P=0.100

P<0.001

P=0.001

P=0.371

P=0.120

A

Figure S6. The maternal FT4 concentration and FTT4P during pregnancy between euthyroid women with and without newborns complicated congenital heart defects by gestational age at the thyroid function test (<12, 12-18, or >18 weeks).

Abbreviations: FT4, free thyroxine; FTT4P, free to total thyroxine proportion.

FT4 (panel A) and FTT4P (panel B) are shown.

P values for the Mann-Whitney U test.
